# Molecular architecture of bacterial type IV secretion systems

**DOI:** 10.1371/journal.ppat.1010720

**Published:** 2022-08-11

**Authors:** Michael J. Sheedlo, Melanie D. Ohi, D. Borden Lacy, Timothy L. Cover

**Affiliations:** 1 Department of Pharmacology, University of Minnesota, Minneapolis, Minnesota, United States of America; 2 Department of Cell and Developmental Biology, University of Michigan, Ann Arbor, Michigan, United States of America; 3 Life Sciences Institute, University of Michigan, Ann Arbor, Michigan, United States of America; 4 Department of Pathology, Microbiology, and Immunology, Vanderbilt University Medical Center, Nashville, Tennessee, United States of America; 5 Veterans Affairs Tennessee Valley Healthcare System, Nashville, Tennessee, United States of America; 6 Department of Medicine, Vanderbilt University School of Medicine, Nashville, Tennessee, United States of America; University of Basel, SWITZERLAND

## Abstract

Bacterial type IV secretion systems (T4SSs) are a versatile group of nanomachines that can horizontally transfer DNA through conjugation and deliver effector proteins into a wide range of target cells. The components of T4SSs in gram-negative bacteria are organized into several large subassemblies: an inner membrane complex, an outer membrane core complex, and, in some species, an extracellular pilus. Cryo-electron tomography has been used to define the structures of T4SSs in intact bacteria, and high-resolution structural models are now available for isolated core complexes from conjugation systems, the *Xanthomonas citri* T4SS, the *Helicobacter pylori* Cag T4SS, and the *Legionella pneumophila* Dot/Icm T4SS. In this review, we compare the molecular architectures of these T4SSs, focusing especially on the structures of core complexes. We discuss structural features that are shared by multiple T4SSs as well as evolutionary strategies used for T4SS diversification. Finally, we discuss how structural variations among T4SSs may confer specialized functional properties.

## Overview of bacterial type IV secretion systems

Bacteria use multiple types of secretion systems to translocate molecules across the cell envelope [1–4]. Type IV secretion systems (T4SSs) are a functionally heterogeneous group of nanomachines that can deliver bacterial proteins and DNA into a diverse range of target cells, including eukaryotic cells (mammalian, plant, insect, and protozoa) and other bacteria [1,2,5–12]. T4SSs can be classified into 2 main groups: conjugation systems and effector translocation systems. Conjugation systems are specialized for transferring mobile genetic elements between bacteria [1,2,5–8]. Effector translocation systems deliver effector proteins, resulting in functional alterations in the target cells [1,2,5–8]. Some T4SSs (exemplified by the *Legionella pneumophila* Dot/Icm T4SS) can deliver several hundred different effector proteins into target cells [13,14]. T4SSs in *Xanthomonas citri* and *Stenotrophomonas maltophilia* deliver effector proteins into other bacteria, resulting in bacterial killing [15–17]. Some T4SSs can deliver bacterial DNA into eukaryotic cells [18,19]. For example, the *Agrobacterium tumefaciens* VirB/VirD4 T4SS translocates a T-DNA-relaxase complex and effector proteins into plant cells, resulting in tumor formation (crown gall disease) [20]. Other T4SSs have the capacity to export DNA or proteins into the extracellular environment (independent of contact with target cells) or import DNA [9]. T4SSs have been investigated mainly in gram-negative bacteria, but these secretion systems are also present in gram-positive bacteria and Archaea [6,9–12].

T4SS-mediated delivery of effector proteins into host cells contributes to the pathogenesis of numerous infections affecting humans [11]. For example, the *Helicobacter pylori* Cag T4SS has an important role in the pathogenesis of gastric cancer and peptic ulcer disease [21–23]. The *L*. *pneumophila* Dot/Icm T4SS is required for the pathogenesis of Legionnaire’s disease [14], and the *Bordetella pertussis* Ptl T4SS has a key role in the pathogenesis of pertussis (whooping cough) [24]. T4SSs also have important roles in the pathogenesis of infections caused by *Bartonella*, *Brucella*, *Coxiella*, *Rickettsia*, *Ehrlichia*, and *Anaplasma* [11].

The first insights into the structural organization of T4SSs came from studies of conjugation systems and the *A*. *tumefaciens* VirB/VirD4 system [7,25–27]. These prototype systems contain 12 components (designated VirB1-11 and VirD4) and are known as “minimized T4SSs” [8]. By convention, the term “minimized T4SS” refers to any T4SS in which the number of components is similar to the number in prototype VirB/VirD4 systems [8]. Some bacteria assemble “expanded T4SSs,” which are more complex than the minimized T4SSs [8]. Examples of expanded T4SSs include the *H*. *pylori* Cag T4SS, the *L*. *pneumophila* Dot/Icm T4SS, and the F plasmid-encoded T4SS. T4SSs in gram-negative bacteria have been classified into type IVa and type IVb subfamilies based on phylogenetic analysis of the sequences of components [12]. This phylogenetic classification does not mirror the classification of T4SSs into minimized and expanded categories. T4SSs in gram-positive bacteria, which typically have fewer components than T4SSs in gram-negative bacteria, have been classified in a type IVc subfamily [28,29].

Recent studies have provided important insights into the molecular architecture of T4SSs. In this review, we compare the macromolecular structures of T4SSs from multiple gram-negative bacterial species. We discuss features that are conserved among T4SSs as well as evolutionary strategies used for T4SS diversification. Finally, we discuss how structural variations among T4SSs may confer specialized functional properties.

## Methods for structural analysis of T4SSs

An important first step in understanding the mechanisms of action of large molecular machines is defining their molecular architecture. Until recently, the only experimental approaches that consistently yielded high-resolution structures were X-ray crystallography and nuclear magnetic resonance (NMR) spectroscopy, which are not well-suited for analyzing the structures of large subassemblies. Technological developments in the field of cryo-electron microscopy (cryo-EM) now allow this technique to routinely determine structures at resolutions amenable to building molecular models directly into the EM density maps. Since EM does not require proteins to pack into crystals and can also be used with heterogenous and dynamic samples, this method can tackle problems that cannot be easily addressed by crystallography or NMR.

New technological advances have also expanded the reach of cryo-EM to reveal structures of complexes in their native cellular environment. Cryo-electron tomography (cryo-ET) allows for the visualization of molecular complexes in the context of vitrified intact cells. When cryo-ET is combined with image analysis approaches such as sub-tomogram averaging, it is possible to attain sub-nanometer resolution of large cellular complexes. Synergy between X-ray crystallography, NMR, single-particle cryo-EM, and cryo-ET has led to an exciting period in the field, yielding important advances in our understanding of the molecular architecture of bacterial secretion systems [30,31].

## Structural organization of T4SSs in gram-negative bacteria

**Fig 1:**
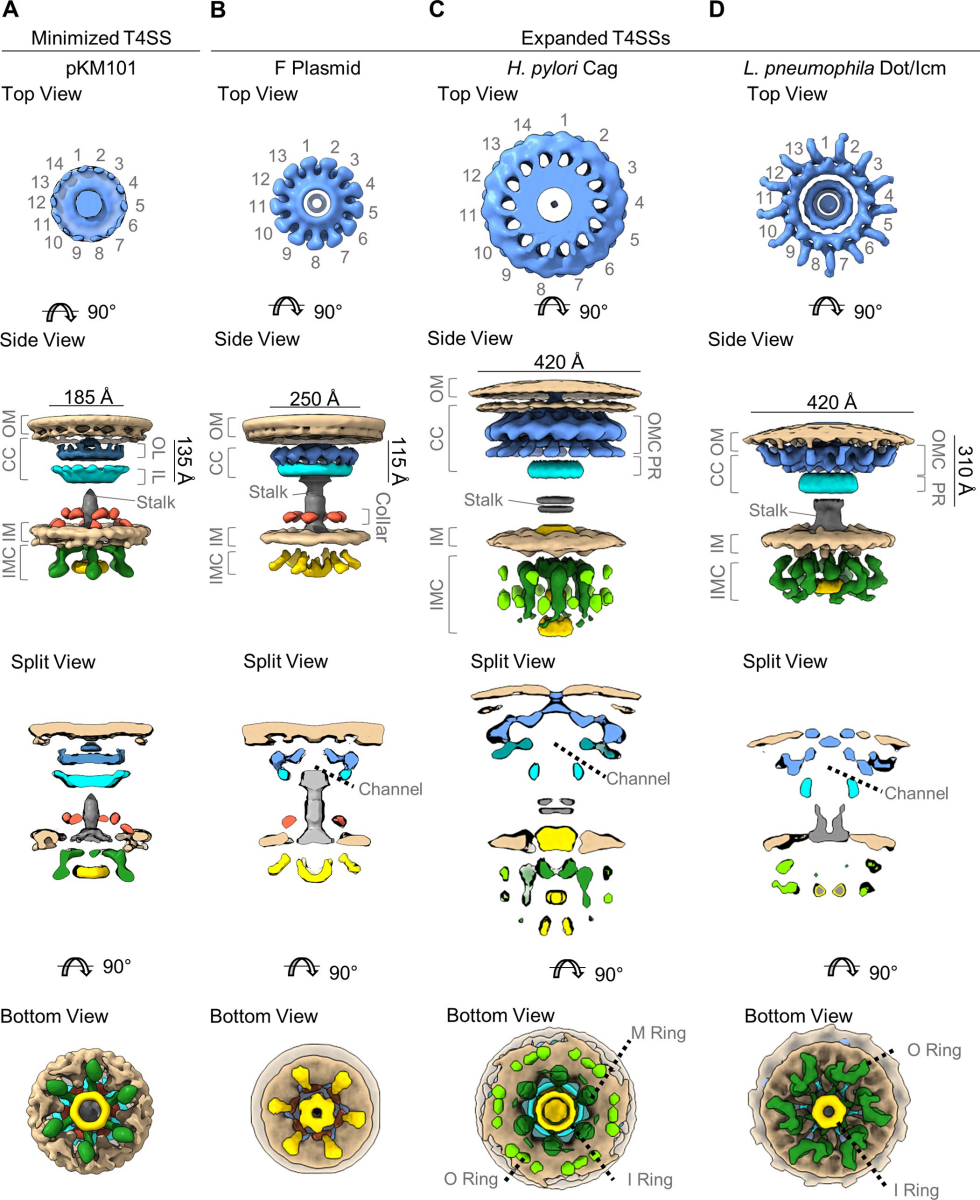
Cryo-ET analyses of T4SSs in situ. Images are shown (left to right) for the pKM101 T4SS (A, EMDB 24098 and 24100), the F plasmid-encoded T4SS (B, EMDB 9344 and 9347), the *H*. *pylori* Cag T4SS (C, EMDB 0635 and 0634), and the *L*. *pneumophila* Dot/Icm T4SS (D, EMDB 7611 and 7612) [32,33,35,37]. Multiple views are shown for each T4SS. The “top” views are relative to the outer membrane (looking into the cell toward the inner membrane). The “bottom” views are relative to the inner membrane (looking out of the cell toward the outer membrane). T4SS structural features include a core complex (CC), stalk (shown in gray), collar (orange), and IMC (shown in green and yellow). The images also show the locations of the OM and IM (tan color). Within the pKM101 and F plasmid-encoded T4SSs, the core complex contains an OL (shown in blue) and an IL (shown in cyan). The *H*. *pylori* Cag and *L*. *pneumophila* Dot/Icm T4SS core complexes contain an OMC (blue) and a PR (cyan). Features within the IMC include O, M, and I rings. cryo-ET, cryo-electron tomography; I, inner; IL, inner layer; IM, inner membrane; IMC, inner membrane complex; M, middle; O, outer; OL, outer layer; OM, outer membrane; OMC, outer membrane cap; PR, periplasmic ring; T4SS, type IV secretion system.

Cryo-ET studies of intact bacteria have provided insights into the overall molecular architecture of T4SSs and spatial relationships with the bacterial cell envelope (outer membrane, inner membrane, and peptidoglycan). The components of T4SSs in gram-negative bacteria are organized, at a minimum, into an outer membrane core complex (subsequently denoted simply as “core complex”) and an inner membrane complex (IMC) (Fig 1) [7,8,32–39]. The core complex is a large structure localized within the periplasm, anchored to the outer membrane. The IMC, composed of proteins anchored to the inner membrane, includes subassemblies that project into the periplasm and others that extend into the cytoplasm. The core complex and IMC are connected by a thin stalk-like structure. Cytoplasmic portions of the IMC include components required for energy generation (ATPases) and substrate recruitment to the T4SS [7,8]. Several features of the core complex and IMC are conserved among T4SSs from different bacterial species, but there are also unique species-specific features, including variation in the overall size of the complexes, differences in the number of components, and differences in the symmetries of subassemblies. Pilus structures, extending from the outer membrane into the extracellular space, are components of some T4SSs but not others [7,8,32,33,40,41].

Thus far, the structures of 4 different T4SSs have been analyzed in intact bacteria using cryo-ET (Fig 1) [32–39]. One of these, the conjugative T4SS encoded by the pKM101 T4SS, is classified as a minimized T4SS, and the other 3 (F plasmid-encoded T4SS, *H*. *pylori* Cag T4SS, and the *Legionella* Dot/Icm T4SS) are classified as expanded T4SSs [8]. The *H*. *pylori* Cag T4SS delivers the CagA effector protein (a putative bacterial oncoprotein) into gastric cells, and it also facilitates entry of LPS metabolites, peptidoglycan, and DNA into gastric cells [21–23]. The *Legionella* Dot/Icm T4SS secretes several hundred effector proteins, thereby facilitating growth of *Legionella* within protozoa or macrophages [13,14].

Cryo-ET analysis of the minimized T4SS encoded by the pKM101 T4SS revealed a core complex that contains an outer and inner layer (O- and I-layer, respectively) [32] (Fig 1A). The O-layer exhibits 14-fold symmetry. “Stalk” structures extending from the core complex toward the inner membrane were visualized in association with about half of the core complexes [32]. Cryo-ET also allowed visualization of the IMC, which includes a periplasmic “collar” with 6-fold symmetry surrounding the base of the stalk, as well as a portion projecting into the cytoplasm [32]. The main component of the cytoplasmic complex is a VirB4-like ATPase, which assembles as a hexamer of dimers (Fig 1A). Finally, pKM101-encoded pilus structures were visualized, either in association with core complexes or emanating from the outer membrane at sites without adjacent core complexes [32].

Cryo-ET visualization of the F plasmid-encoded T4SS revealed many features similar to those of the pKM101 T4SS, including a core complex, a cylinder or stalk connecting the core complex to the IMC, and a “collar” encircling the base of the cylinder (adjacent to the inner membrane) (Fig 1B) [33]. The portion of the core complex adjacent to the outer membrane has 13-fold symmetry (13 visible spokes). The IMC has 6-fold symmetry and includes a cytoplasmic portion characterized by a hexamer of dimers. A central channel is present within both the core complex and the IMC. Superfolder GFP (sfGFP) fused to the VirB4-like ATPase was detectable as extra densities associated with the central hexamer of dimers [33]. Cryo-ET allowed visualization of complete T4SSs in which the F-pilus, core complex, and IMC were all intact and contiguous [33]. Multiple additional forms of the T4SS apparatus, hypothesized to represent different stages of assembly or disassembly, were also seen [33].

Cryo-ET analysis of the *H*. *pylori* Cag T4SS in intact bacteria (Fig 1C) revealed a core complex much larger in size than corresponding core complexes in the pKM101 or F plasmid-encoded T4SSs (Fig 1A) [34,35]. The portion of the core complex adjacent to the outer membrane has 14-fold symmetry. Radial spokes are present in 2 layers of the core complex, and interestingly, the spokes of the 2 layers are arranged with opposite chirality (clockwise or counterclockwise) [35]. A large central channel is visible within the *H*. *pylori* Cag T4SS core complex. A “collar” encircling the central cylinder or stalk at the base of the core complex was visualized, as well as a “plug” within the core complex. Core complexes were not visible in CagX, CagY, or CagM mutant strains, which suggest these 3 proteins are required for core complex assembly [35]. Cryo-ET analysis also allowed visualization of an IMC with 6-fold symmetry. The cytoplasmic portion of the IMC is organized as 3 concentric rings, termed the inner (I), middle (M), and outer (O) rings, surrounding a central channel (Fig 1C). The IMCs of mutant strains lacking Cagα (VirB11 homolog), Cagβ (VirD4 homolog), and CagE (VirB4 homolog) exhibited detectable differences compared to the IMCs of wild-type *H*. *pylori*, consistent with the localization of these proteins to the IMC [35].

Cryo-ET studies of vitrified *L*. *pneumophila* showed the presence of a large T4SS core complex, similar in size compared to the *H*. *pylori* Cag T4SS core complex but configured as a wheel or disk-like structure (Fig 1D) [36–39]. The portion of the core complex adjacent to the outer membrane has 13-fold symmetry (13 visible spokes), and the IMC has 6-fold symmetry (Fig 1D) [37,38]. Interestingly, a Dot/Icm core complex can be assembled in the absence of DotG (VirB10 homolog) [38]. The cytoplasmic portion of the IMC is predicted to contain a hexameric assembly of DotO (VirB4) dimers, DotB (VirB11), and additional proteins (Fig 1D). Localization of DotB (VirB11 homolog) to the IMC was detected by fusing sfGFP to a stably docked mutant form of DotB [37]. A current model proposes that DotB dynamically engages and disengages from DotO. Multiple distinct subassembly intermediate forms of the Dot/Icm T4SS have been detected, along with conformational variations in the IMC. Specifically, docking of DotB onto the DotO complex promotes a conformational change in the secretion system associated with opening of an inner membrane channel [39].

T4SS-associated pili have been visualized by cryo-ET in studies of both the F-type T4SS and the pKM101-encoded T4SS [32,33]. Thus far, there have not been any reports of pili associated with the *L*. *pneumophila* Dot/Icm T4SS. Several publications reported visualization of filamentous structures produced by *H*. *pylori* and proposed that these are pilus components of the Cag T4SS [34,42–44]. Cryo-ET analysis of *H*. *pylori* cocultured with gastric epithelial cells detected membranous tubes extending from the bacterial surface [34], and these were proposed to be pilus components of the Cag T4SS. Notably, the tube-like structures visualized by cryo-ET were never detected in direct proximity to the core complex. Similar outer membrane extensions have been visualized by cryo-ET in many different bacterial species, without a requirement for contact with mammalian cells [45]. At present, it remains unclear if the *H*. *pylori* filaments or tubes are pilus-like components of the *H*. *pylori* Cag T4SS or structures unrelated to the Cag T4SS.

The resolution of cryo-ET analyses thus far has been limited to about 3 nm, which is not sufficient for high-resolution delineation of protein structures and protein–protein interactions. Alternate methods have been used to attain atomic level resolution of core complexes, IMC components, and pili, as discussed in the following sections.

## Structural features of core complexes from minimized T4SSs

High-resolution structures of subassemblies or individual components of minimized T4SSs have been generated by using either X-ray crystallography or single-particle cryo-EM methods. Early studies of protein–protein interactions among components of T4SSs suggested that several VirB proteins could assemble into one or more protein complexes [46]. Subsequently, 4 genes in the pKM101 conjugative plasmid [encoding TraN (VirB7), TraE (VirB8), TraO (VirB9), and TraF (VirB10)] were cloned and expressed, and a complex containing VirB7, VirB9, and VirB10 (but not VirB8) was isolated [47]. Cryo-EM analysis of the isolated particles revealed a barrel-shaped structure about 185 Å in height and diameter with 14-fold symmetry [47], corresponding to the pKM101 core complex visualized by cryo-ET [32]. Outer and inner layers (O- and I-layers) could be distinguished [47], similar to the organization visualized by cryo-ET (Fig 1) [32].

**Fig 2:**
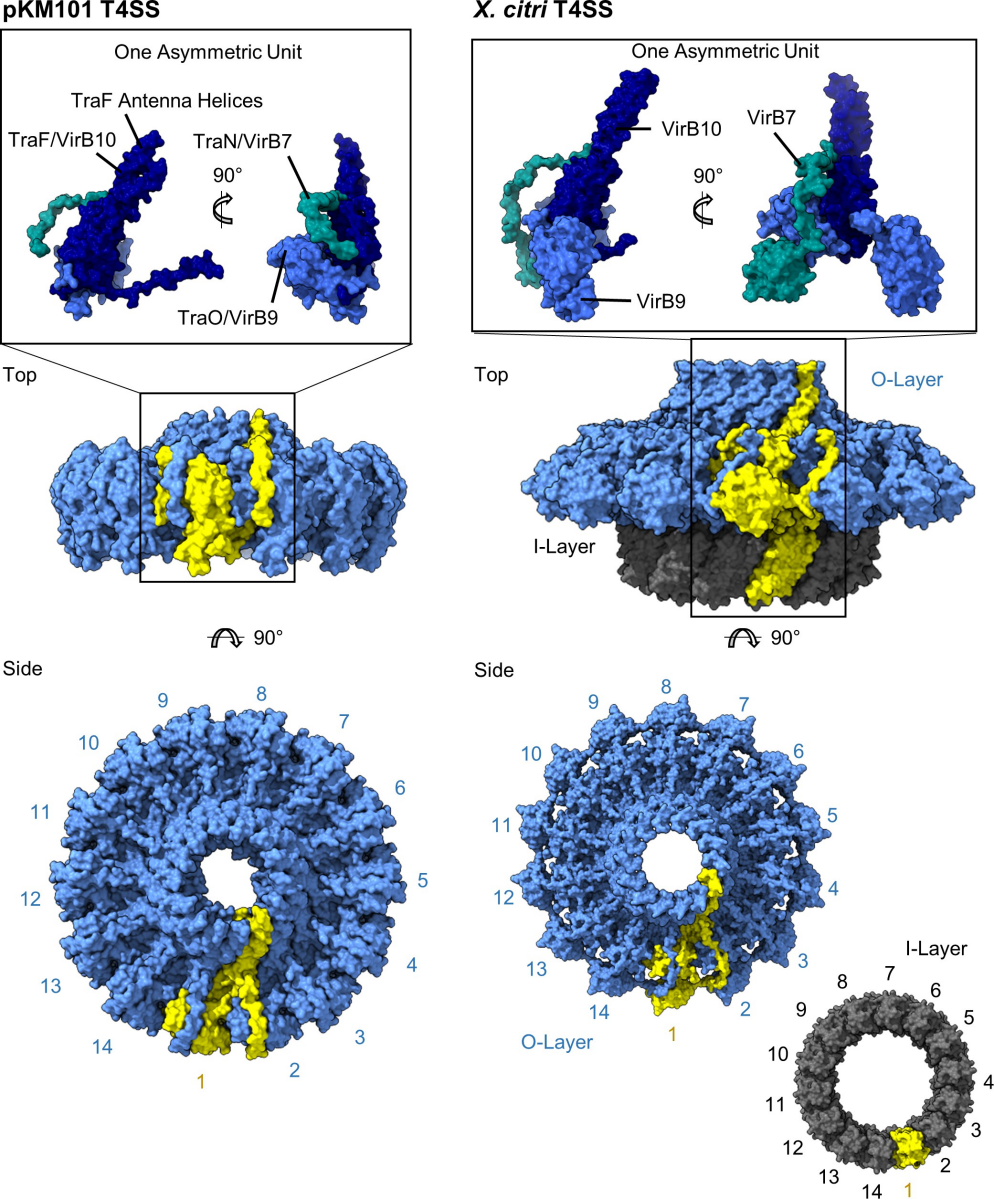
Structure of core complexes from minimized T4SSs. The structures of core complexes from the pKM101 (PDB 3JQO) and *X*. *citri* (PDB 6GYB) T4SSs are shown as surface representations [48,52]. The O-layers are shown in blue, and the I-layer of the *X*. *citri* core complex is shown in gray. The asymmetric unit for each T4SS is indicated in yellow and consists of VirB7, VirB9, and VirB10. The structures of the asymmetric unit for each system are shown in the inset. T4SS, type IV secretion system.

Chymotryptic cleavage of the purified core complex yielded the isolated O-layer, which contains the C-terminal portion of VirB9 (TraO), the C-terminal portion of VirB10 (TraF), and the full-length VirB7 (TraN) [48]. A crystal structure of the isolated O-layer showed that VirB10 is localized in the center, surrounded by VirB9 and VirB7. A central ring, composed of 14 two-helix bundles of VirB10 α-helices (designated as the “antenna region”), was proposed to form a channel through the outer membrane (Fig 2). In support of this hypothesis, an epitope tag inserted between VirB10 α-helices in the antenna region was detected extracellularly [48]. The I-layer is composed of N-terminal domains of VirB9 and VirB10 [49].

Further insights were provided by a study in which 10 genes in the R388 conjugative plasmid (*virB1-10*) were cloned and expressed [50]. Eight of the encoded proteins (VirB3-VirB10) assembled into a protein complex. EM analysis of the purified R388-encoded T4SS assembly revealed a core complex connected to a putative IMC by a thin stalk. It was proposed that VirB10 forms a connection between the core complex and the IMC. The IMC, predicted to be composed of the VirB4 ATPase, was organized into 2 side-by-side hexameric barrels [50]. Analysis of similar complexes containing VirD4 (an ATPase thought to be responsible for substrate recruitment) showed that VirD4 dimers are localized between the VirB4 ATPases [51]. Notably, the structural organization of the IMC visualized in the isolated T4SS particles [50] differed from that of the pKM101 IMC visualized by cryo-ET [32]. Despite this difference, both the cryo-ET analysis and analysis of isolated T4SS particles showed that the IMC has a molecular organization different from that of the core complex (6-fold and 14-fold symmetry, respectively) [32,50,51].

Like pKM101- and R388-encoded T4SS core complexes, core complexes within the *X*. *citri* and the *A*. *tumefaciens* VirB/VirD4 T4SSs are also composed of VirB7-, VirB9-, and VirB10-like proteins [52,53]. In contrast to the barrel-like shape of the pKM101 core complex, the *X*. *citri* core complex has a “flying saucer” shape (Fig 2) [52]. This feature of the *X*. *citri* T4SS is at least partially attributable to the presence of an additional domain in *X*. *citri* VirB7. High-resolution structural analysis of the *X*. *citri* core complex revealed features of the I-layer (composed of N-terminal portions of VirB9 and VirB10) that had not been resolved in the previous studies of conjugation systems [52]. Collectively, the analyses of these minimized T4SSs indicate that they all contain core complexes composed of VirB7, VirB9, and VirB10, organized into 2 ring-like layers (O-layer and I-layer) with 14-fold symmetry (Fig 2).

## Structural features of core complexes from expanded T4SSs

**Fig 3:**
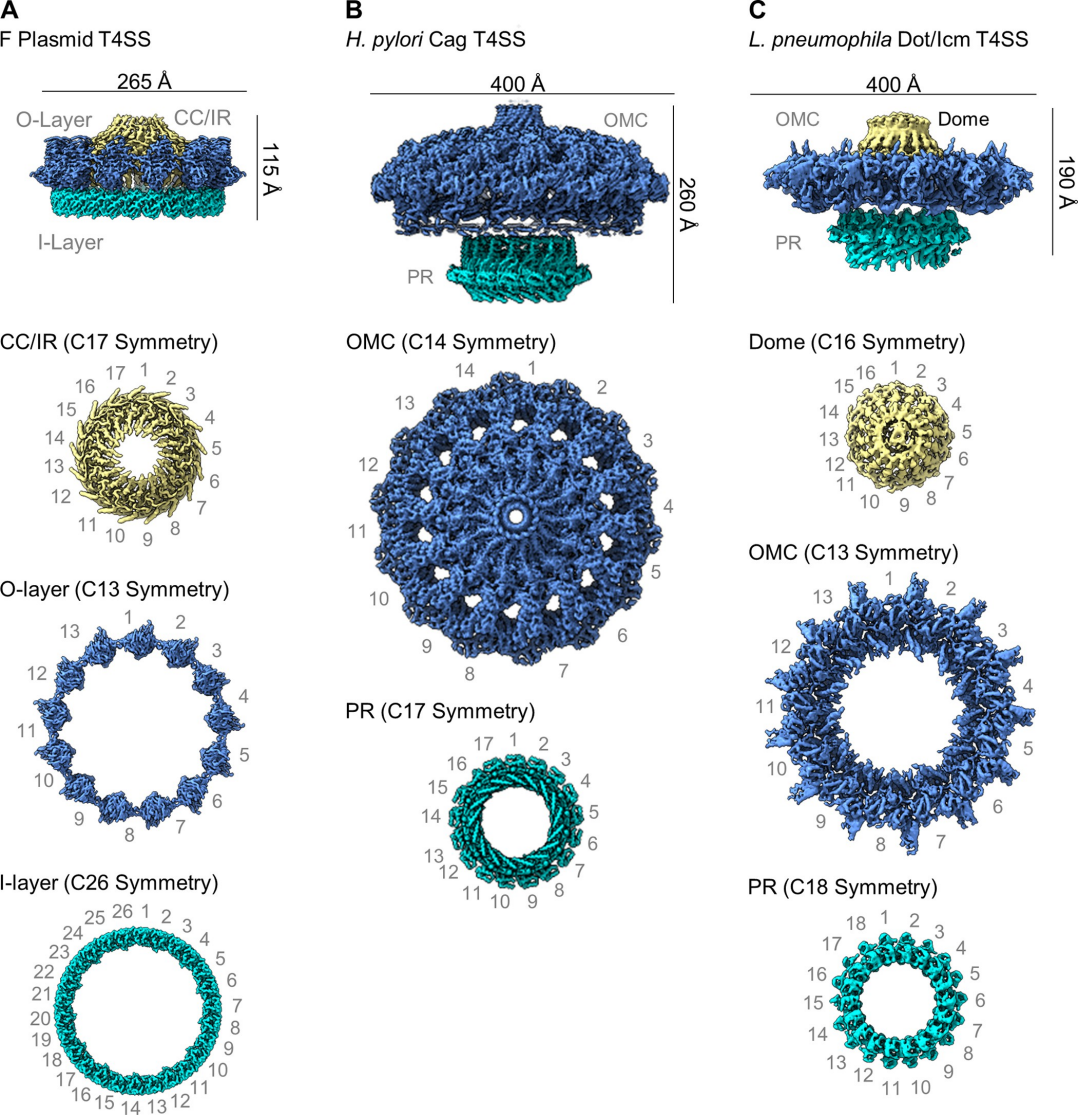
Overall architecture of core complexes in expanded T4SSs. The structure of each expanded T4SS core complex was determined in 2 or 3 parts, corresponding to regions with different symmetries [56,57,60,61]. The F plasmid T4SS core complex is characterized by a CC or IR (yellow), and an outer (peripheral) ring comprised of an O-layer (blue) and I-layer (green) (EMDB 12962, 12963, and 13231). OMC and PR regions (blue and green) are distinguishable in the *H*. *pylori* Cag T4SS core complex (EMDB 20021 and 22081). The *L*. *pneumophila* Dot/Icm T4SS core complex contains 3 features termed a dome (yellow), disk (blue), and PR (green) (EMDB 24018, 24005, and 24006). The figure illustrates symmetry mismatch between adjacent regions. CC, central cone; IR, inner ring; OMC, outer membrane cap; PR, periplasmic ring; T4SS, type IV secretion system.

Two independent studies have reported the structure of F plasmid (pED208)-encoded T4SS core complexes [54,55]. In the first study, the genes encoding 3 component proteins (TraV, TraK, and TraB, corresponding to VirB7, VirB9, and VirB10, respectively) were cloned and overexpressed in *E*. *coli*, and the resulting complex was purified and analyzed by cryo-EM [54]. In the second study, core complexes were purified from an *E*. *coli* strain containing the entire pED208 plasmid [55]. The overall dimensions of F plasmid-encoded core complexes are 115 Å in height and 265 Å in diameter (Fig 3A). The F plasmid-encoded core complex contains a central channel, about 125 Å in diameter [54,55]. Consistent with the organization of minimized T4SSs, both an O-layer and an I-layer are present in the F-type core complex (Fig 3A). The F plasmid-encoded core complex is organized as 2 radial concentric rings exhibiting a symmetry mismatch (13-fold symmetry in the peripheral portion of the O-layer and 17-fold symmetry in the central portion of the O-layer) (Fig 3A) [54,55]. Portions of TraV and TraB are present in both the central and peripheral rings [54,55]. Within the peripheral ring, the O-layer exhibits 13-fold symmetry, and the I-layer exhibits 26-fold symmetry. The entire complex contains a total of 69 polypeptide chains: 17 copies of TraB, 26 copies of TraK, and 26 copies of TraV [54]. Thus, the core complex of the F plasmid-encoded T4SS is assembled from 3 building blocks, but its architecture is more complex than that of core complexes in minimized T4SSs because of the duplication of VirB7 and VirB9 components and symmetry mismatch between the 2 radial concentric rings.

**Fig 4:**
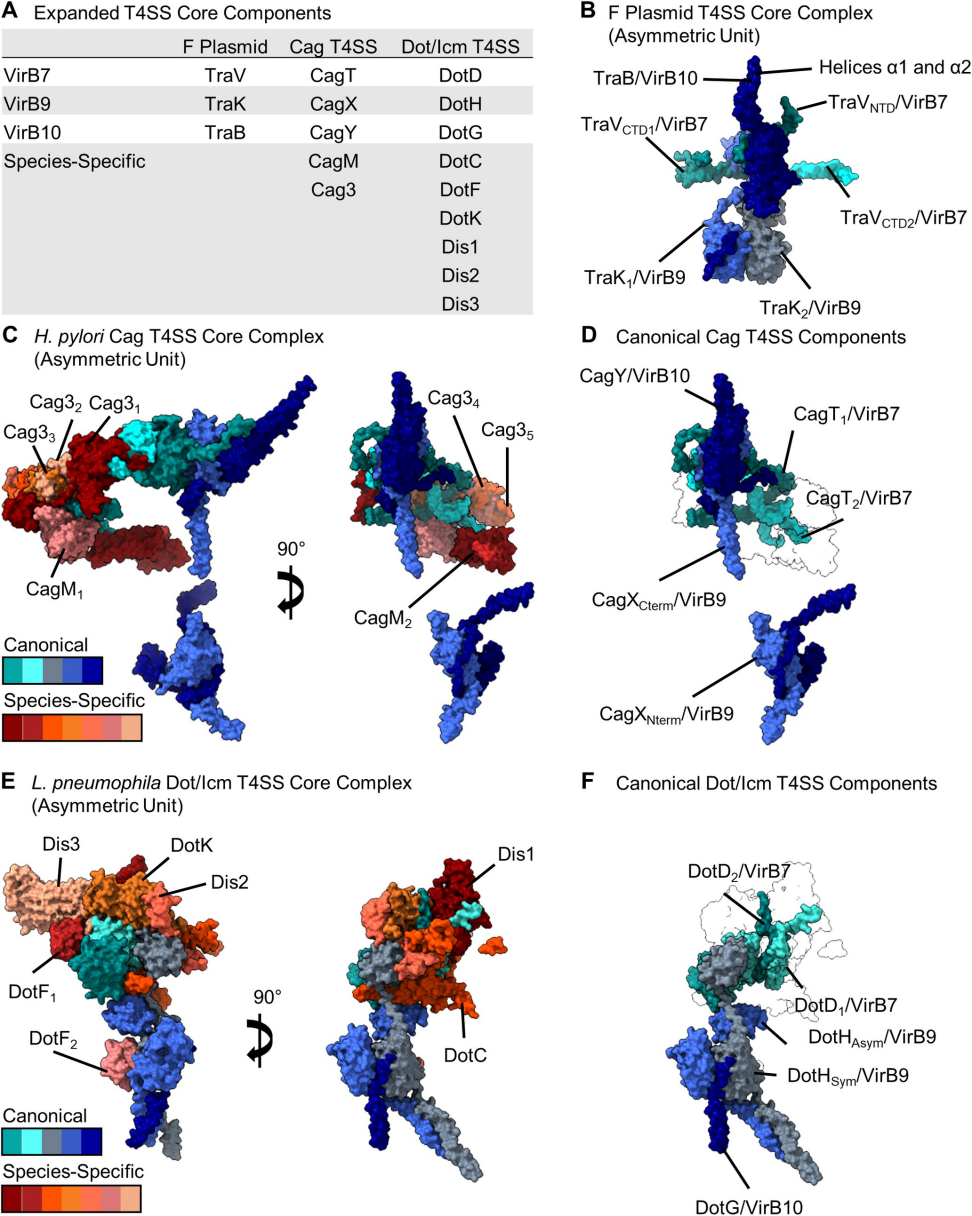
Components of core complex asymmetric units in expanded T4SSs. (A) Listing of core complex components in 3 expanded T4SSs. (B) Asymmetric unit of the core complex in the F plasmid-encoded T4SS shown in surface representation (PDB 7OKO and 7OKN) [54,55]. (C) Asymmetric unit of the core complex in the *H*. *pylori* Cag T4SS shown in surface representation (PDB 6X6S and 6X6J) [56,57]. Components that are related to canonical T4SS proteins (CagT, CagX, and CagY) are shown in shades of blue/green, and species-specific components (CagM and Cag3) are depicted in shades of red. (D) Structures of the VirB7, VirB9, and VirB10-like proteins in the *H*. *pylori* Cag asymmetric unit. (E) Asymmetric unit of the core complex in the *L*. *pneumophila* Dot/Icm T4SS shown in surface representation (PDB 7MUC) [60,61]. Components that are related to canonical T4SS proteins (DotD and DotH) are shown in shades of blue/green, and species-specific components (DotC, DotF, DotK, Dis1, Dis2, and Dis3) are depicted in shades of red. (F) VirB7, VirB9, and VirB10-like proteins in the *L*. *pneumophila* Dot/Icm asymmetric unit. T4SS, type IV secretion system.

Single-particle cryo-EM studies of Cag T4SS core complexes extracted from *H*. *pylori* revealed large particles with a mushroom-shaped structure, about 400 Å in width, with a hollow interior (Fig 3B) [56,57]. These core complexes contain 5 different proteins: CagY (VirB10 homolog), CagX (VirB9 homolog), CagT (VirB7 homolog), CagM, and Cag3 [56–58] (Figs 3B and 4C). The latter 2 proteins are species-specific components that lack sequence relatedness to other known proteins. Three main regions of the *H*. *pylori* Cag T4SS core complex have been described: an outer membrane cap (OMC), a periplasmic ring (PR), and a stalk [56,57]. The OMC has 14-fold symmetry, and the PR has 17-fold symmetry (Fig 3B) [56,57]. Two distinct layers of the OMC of the Cag T4SS have been described, termed the O-layer and the I-layer [56,57]. The I-layer of the Cag T4SS has structural features that are distinct from those of I-layers in minimized T4SSs. The OMC contains all 5 core complex components (CagY, CagX, CagT, CagM, Cag3) in a 1:1:2:2:5 stoichiometric ratio (i.e., 14 copies of CagY and CagX, 28 copies of CagT and CagM, and 70 copies of Cag3, for a total of 154 components) [56,57]. The PR is composed of CagY and CagX in a 1:1 ratio (i.e., 17 copies of both CagY and CagX) [57]. Distinct domains of CagX and CagY are localized within the OMC (CagX residues 349–514 and CagY residues 1677–1909) and the PR (CagX residues 32–325 and CagY residues 1469–1603). Analysis of the cryo-EM maps allowed for visualization of CagX connections between the PR and the OMC and suggested that 3 copies of CagY and CagX are incorporated into the 17-fold symmetric PR but excluded from the 14-fold symmetric OMC, resulting in the observed symmetry mismatch [57]. A stalk at the base of the core complex was visualized but not well resolved in cryo-EM studies [56]. The composition and symmetry of the stalk have not yet been defined.

Although *H*. *pylori* CagY, CagX, and CagT exhibit sequence relatedness to VirB10, VirB9, and VirB7 homologs in other bacterial species, they also exhibit unique features. Most notably, these 3 *H*. *pylori* proteins are much larger in size than the corresponding proteins in minimized systems and contain unique domains. At a sequence level, Cag3 and CagM are unrelated to any other known bacterial proteins, but interestingly, the Cag3 structure resembles that of CagT [57]. The large size of the *H*. *pylori* Cag T4SS core complex is attributable to incorporation of multiple copies of CagT, CagM, and Cag3 into each asymmetric unit, as well as the relatively large sizes of CagY, CagX, and CagT proteins compared to corresponding VirB10, VirB9, and VirB7 proteins in minimized T4SSs.

Single-particle cryo-EM analyses showed that the isolated Dot/Icm T4SS core complexes extracted from *L*. *pneumophila* are about 400 Å in width and 190 Å in length/height [59–61], similar to the size of the *H*. *pylori* Cag T4SS core complex, and contain a large central cavity (Fig 3C). Two main regions of the Dot/Icm core complex have been described (OMC and PR) [60,61], which resembles the organization of the *H*. *pylori* Cag core complex. The Dot/Icm OMC is characterized by a central dome and a flat disk with 13 arms [60,61]. Multiple sites of symmetry mismatch were observed within the Dot/Icm core complex: 16-fold symmetry of the OMC dome, 13-fold symmetry of the OMC disk, and 18-fold symmetry of the PR [61]. Distinct inner and outer layers of the OMC disk were not detected, and a stalk was not visualized.

Early studies provided evidence that the *Legionella* Dot/Icm core complex is composed of at least 5 different proteins: DotD, DotH and DotG (corresponding to VirB7, VirB9, and VirB10, respectively), and 2 species-specific components (DotF and DotC) [59,62]. Single-particle cryo-EM analysis of isolated core complex particles confirmed that these 5 proteins are present, along with at least 4 additional constituents: DotK and 3 proteins not previously known to be associated with the T4SS (now termed **D**ot/**I**cm **s**ecretion proteins Dis1, Dis2, and Dis3) (Fig 4E and 4F) [60,61]. Two copies of DotD and 1 copy of the other proteins are present within the asymmetric unit. The PR is composed of 4 proteins, including a second copy of DotF (called DotF_2_), DotG (a VirB10 homolog), DotH (a VirB9 homolog), and another protein whose identity has not yet been determined [60,61]. Thirteen linkers corresponding to portions of DotH were detected between the OMC and PR. Five copies of DotH detected within the PR do not span the symmetry mismatch between the OMC (13-fold symmetry) and the PR (18-fold symmetry) [61]. Therefore, the VirB9-like DotH protein forms part of both the OMC and PR and bridges the symmetry mismatch, an organization resembling that observed with the *H*. *pylori* Cag T4SS. Overall, the Dot/Icm core complex contains a larger number of components and increased structural complexity compared to core complexes in any of the other T4SSs analyzed thus far.

## Structural analyses of inner membrane complexes and F pili

In contrast to T4SS core complexes, which are relatively stable in detergent, intact IMCs have been difficult to purify, hindering high-resolution structural studies. Nevertheless, the structures of IMC subassemblies or individual IMC components have been successfully determined, using either cryo-EM or crystallization methods [63,64]. IMCs from minimized T4SSs are predicted to contain multiple ATPases (VirB4, VirB11, and VirD4) along with several other components (including VirB3, VirB5, VirB6, and VirB8) [50]. VirD4 plays a critical role in recruitment of effector proteins [7,8,65,66]. High-resolution structural models have been determined for the *L*. *pneumophila* Dot/Icm coupling complex, which functions to recognize and recruit effector proteins. The coupling complex contains DotL (a VirD4-like ATPase) and multiple additional proteins (including DotM, DotN, DotY, DotZ, lcmS, IcmW, and LvgA) [67–70]. The approximately 300 kDa mass of the complex suggests that it contains 1 copy of each component [69]. It is predicted that the approximately 300 kDa complexes assemble into larger hexameric subassemblies within the IMC.

Conjugative pili have an important role in horizontal transfer of DNA among bacteria. F plasmid-encoded pili have been extensively studied and are characterized by an ability to dynamically extend and retract [8,33,40]. Cryo-EM studies showed that these structures are composed of thousands of copies of the TraA (VirB2) pilin bound to phospholipid [71,72]. The TraA-phospholipid building blocks of several T4SS pilus systems are organized as pentameric complexes in a helical assembly [71], whereas the building blocks of other T4SS pilus systems have a non-pentameric organization [72].

## Conserved structural features of T4SSs

Minimized T4SSs can translocate DNA and proteins across the cell envelope, and accordingly, many structural features of minimized T4SSs are conserved among the expanded T4SSs. The core complex is presumed to be one of the first structures assembled during T4SS biogenesis [35,38]. Proteins resembling VirB7, VirB9, and VirB10 are present in nearly all the T4SSs analyzed thus far and can be viewed as the fundamental building blocks for core complex assembly. VirB10-like proteins are predicted to span from the inner membrane to the outer membrane, and these proteins constitute the central portion of the core complex. A VirB10 “antenna region” is positioned at the apex of core complexes in all T4SSs analyzed thus far and is predicted to form an outer membrane channel [48]. The VirB10 antenna region is characterized by a “2-helix bundle ring system” [48]. The α-helical structure of this portion of VirB10 differs from the structures of most outer membrane proteins in gram-negative bacteria, which typically have a β-barrel architecture. The high conservation of C-terminal VirB10 sequences among T4SSs, the central localization of VirB10 within core complexes, and the apparent insertion of VirB10 into the outer membrane are consistent with a key role of VirB10 in core complex assembly and function. While VirB10 is thought to be essential for core complex assembly in many species [35], *Legionella* Dot/Icm core complexes can apparently assemble in the absence of VirB10 [38].

Conserved features of core complex organization include the presence of 2 layers (O- and I-layers) and the presence of radial spokes. The O-layers of core complexes in minimized systems contain VirB7 and C-terminal portions of VirB9 and VirB10, and the O-layers of expanded systems contain related components. The lipid components of the VirB7-like proteins are positioned close to the outer membrane [57]. The I-layers of core complexes in minimized systems contain N-terminal portions of VirB9 and VirB10 [52], and the compositions of the F-type T4SS I-layer, *H*. *pylori* PR, and *Legionella* PR mimic that of the I-layer in minimized systems. There are also similarities in the molecular architecture of the IMCs from multiple T4SSs, including 6-fold symmetry and the presence of multiple ATPases (along with other components).

Symmetry mismatch is a striking feature of T4SSs. All the T4SSs examined thus far exhibit symmetry mismatch at the junction between the IMC and the core complex [32,33,35,37–39,50], and symmetry mismatch is also present within core complexes of expanded T4SSs [54–57,60,61]. Sites of symmetry mismatch are likely to provide regions of mobility or flexibility between adjacent layers. These sites might provide a “ratcheting” mechanism that keeps substrates moving in one direction. Thus far, it has been difficult to detect the proposed mobility between adjacent layers, and the mechanisms by which symmetry mismatch contribute to T4SS function remain poorly understood. Symmetry mismatch has been detected not only within T4SSs but also within several other types of bacterial secretion systems, including T2SSs, T3SSs (and related flagellar systems), and T6SSs [73–77]. The presence of symmetry mismatch within these diverse secretion systems suggests that it has an important functional significance.

## Evolutionary strategies for T4SS diversification

Recent studies of T4SSs from multiple bacterial species provide insight into the range of variation that exists in their molecular architecture. Incorporation of novel components is one of the recurring phenomena that can lead to variations in the architecture of T4SSs. For example, the core complexes of expanded T4SSs from *H*. *pylori* and *L*. *pneumophila* have diameters that are about twice the size of the diameters of core complexes in minimized systems, and this variation is attributed in part to the incorporation of species-specific components. The sequences and structures of the species-specific components in *H*. *pylori* are unrelated to those of the species-specific components in *Legionella*. Therefore, the genes encoding these components in the 2 species were probably acquired through independent horizontal transfer events. Interestingly, the structure of *H*. *pylori* Cag3 resembles that of *H*. *pylori* CagT (a VirB7 homolog), despite a lack of sequence relatedness between Cag3 and CagT [57]. The similarity in structures of Cag3 and CagT may be attributable to convergent evolution. At present, it is not known what benefits are conferred by the enlarged size of *H*. *pylori* and *L*. *pneumophila* T4SS core complex subassemblies. Current evidence suggests that T4SS effector proteins are translocated in an unfolded state [78,79], so the enlarged size is unlikely related to the size of the secreted effector proteins.

Altering the stoichiometry of subassembly components is another mechanism leading to diversification of T4SS architecture. For example, the F-type T4SS core complex is assembled from the same 3 building blocks found in minimized systems, but additional copies of the VirB7 and VirB9 components (TraV and TraK) are observed within the asymmetric unit [54,55] (Fig 4). Similarly, within asymmetric units of the *H*. *pylori* Cag T4SS and *L*. *pneumophila* Dot/Icm core complexes, there are 2 copies of the VirB7 homologs (CagT and DotD, respectively) [57,61] (Fig 4). Thus, incorporation of an increased number of VirB7 and/or VirB9 components is a feature shared by core complexes in multiple expanded T4SSs.

Among T4SS components that are conserved in T4SSs from multiple species, there is a high level of variation in the sequences and structures. Comparative analysis of VirB7, VirB9, and VirB10-like proteins in T4SSs from multiple species provides insight into the ways in which the sequences and structures of these proteins have diversified and how this affects T4SS architecture. One of the recurring features is incorporation of extra domains into these proteins, leading to variations in size. For example, VirB7 in the minimized T4SSs is a small protein (about 55 amino acids in length in *A*. *tumefaciens*), whereas VirB7-like proteins in *H*. *pylori* and *Legionella* are 280 and 163 amino acids in length, respectively. Similarly, VirB10- and VirB9-like proteins are much larger in *H*. *pylori* and *Legionella* T4SSs than in minimized systems. For example, CagY (VirB10 homolog) in *H*. *pylori* contains a large region characterized by amino acid repeat units [80], which is not present in VirB10 proteins in the minimized systems.

There is also considerable variation in the symmetry of elements within core complexes from multiple T4SSs. For example, the O-layers of core complexes in minimized T4SSs have 14-fold symmetry, whereas core complexes from *H*. *pylori*, *Legionella*, and the F-type T4SS have 13-, 14-, 16-, 17-, or 18-fold symmetry in various regions. The functional significance of these variations and the structural determinants of symmetry organization are not yet known. Similarly, it is not known whether the exact geometry of symmetry mismatches is functionally important.

## Specialized functions conferred by specific T4SS structural features

T4SSs exhibit an extraordinary diversity of functions, and recent studies have revealed marked diversity in the molecular architectures of T4SSs. Presumably the specialized structural features of individual T4SSs are linked to their unique functions, but these relationships are not yet understood. As one approach for dissecting how specialized structural features contribute to specific functions, we can consider ways in which the structural organization of expanded T4SSs confers functional properties not exhibited by minimized T4SSs. For example, we speculate that the numerous species-specific components and structural complexity of the *L*. *pneumophila* Dot/Icm system contribute to its ability to secrete several hundred different effector proteins [13,14]. Similarly, we hypothesize that species-specific components and modifications of conserved T4SS components in the *H*. *pylori* Cag T4SS allow it to deliver multiple types of substrates (protein, DNA, lipopolysaccharide metabolites, and peptidoglycan) into host cells [21–23] and alter host cell physiology through translocation-independent mechanisms [81,82]. The organization of the F-type core complex (2 radially concentric rings exhibiting symmetry mismatch) presumably facilitates the processes required for pilus extension and retraction [40], as well as transitions between secretion of pilus components and secretion of plasmid DNA. Experimental studies, coupled with analyses of T4SSs from additional bacterial species, will be required to better understand the relationships between specialized structural and functional features of T4SSs.

## Future prospects

Studies conducted over the past decade have revealed both conserved features and variation in T4SS structural organization. For a more comprehensive view of T4SS diversity, it will be important to analyze T4SSs from additional species. Single-particle cryo-EM and crystallization methodology have been used to obtain high-resolution structural models of isolated T4SS subassemblies or individual proteins, and cryo-ET methods have been used to visualize T4SSs within intact bacteria. Future studies, focused on determining high-resolution structures of T4SS subassemblies in the context of intact membranes, will provide additional information about T4SS structure and function within a membrane environment.

The studies conducted thus far have provided valuable insights into T4SS architecture and composition, but relatively little is known about the process of T4SS assembly, the functional roles of individual T4SS components, or the mechanisms of substrate translocation. In future studies, it will be important to define multiple conformational states of T4SSs, visualize intermediate stages in the processes by which T4SSs are assembled, and trace the path of effector proteins or other substrates through the assemblies. It will be important to define the functions of not only the conserved components of T4SSs but also the functions of species-specific components. Similarly, it will be important to determine the functional consequences of diversification in conserved T4SS components. Defining structural relationships between membrane-spanning T4SS components, pilus components, effector proteins, and other substrates at multiple stages of the translocation process will lead to a better understanding of the mechanisms of substrate translocation. We anticipate that such studies will reveal substantial mobility and conformational changes within the T4SS architecture, including mobility at sites of symmetry mismatch. Finally, we anticipate that the increasing wealth of T4SS structural data will help guide the development of agents that inhibit T4SS function, which might be useful in conjunction with traditional antibiotics for the treatment of bacterial infections.
